# Bone Micro-CT Assessments in an Orchidectomised Rat Model Supplemented with *Eurycoma longifolia*

**DOI:** 10.1155/2012/501858

**Published:** 2012-08-17

**Authors:** Rosmaliza Ramli, Mohd Fadhli Khamis, Ahmad Nazrun Shuid

**Affiliations:** ^1^Department of Pharmacology, Faculty of Medicine, Universiti Kebangsaan Malaysia, 50300, Kuala Lumpur, Malaysia; ^2^School of Dental Sciences, Universiti Sains Malaysia, 16150 Kubang Kerian, Kelantan, Malaysia

## Abstract

Recent studies suggested that *Eurycoma longifolia*, a herbal plant, may have the potential to treat osteoporosis in elderly male. This study aimed to determine the effects of *Eurycoma longifolia* supplementation on the trabecular bone microarchitecture of orchidectomised rats (androgen-deficient osteoporosis model). Forty-eight-aged (10–12 months old) *Sprague Dawley* rats were divided into six groups of sham-operated (SHAM), orchidectomised control (ORX), orchidectomised + 7 mg/rat testosterone enanthate (TEN) and orchidectomised + *Eurycoma longifolia* 30 mg/kg (EL30), orchidectomised + *Eurycoma longifolia* 60 mg/kg (EL60), orchidectomised + *Eurycoma longifolia* 90 mg/kg (EL90). Rats were euthanized following six weeks of treatment. The left femora were used to measure the trabecular bone microarchitecture using micro-CT. Orchidectomy significantly decreased connectivity density, trabecular bone volume, and trabecular number compared to the SHAM group. Testosterone replacement reversed all the orchidectomy-induced changes in the micro-CT parameters. EL at 30 and 60 mg/kg rat worsened the trabecular bone connectivity density and trabecular separation parameters of orchidectomised rats. EL at 90 mg/kg rat preserved the bone volume. High dose of EL (90 mg/kg) may have potential in preserving the bone microarchitecture of orchidectomised rats, but lower doses may further worsen the osteoporotic changes.

## 1. Introduction

*Eurycoma longifolia*, known locally as “*Tongkat Ali*” in Malaysia is a native herb in the Southeast Asia region, especially in Malaysia, Indonesia, Cambodia, Laos, and Vietnam [1]. This small plant from the *Simaroubaceae* family can grow up to more than 15 meters and starts to bear fruits after 2-3 years of cultivation. Since decades ago, almost all parts of this evergreen plant including the fruits, root, and leaves have been sought after for its medicinal uses. However, the most valuable component is the root which has been more commonly used as an aphrodisiac and also to treat a wide range of diseases, including aches, fever, malaria, sexual insufficiency and glandular swelling [2–4]. The root extract of *Eurycoma longifolia* has been used as herbal ingredients to enhance blood flow and restore vitality and energy following childbirth [5]. Due to these therapeutic values, *Eurycoma longifolia* has been commercialized in various forms of health supplement [6], and added to beverages such as coffee and tea. Since decades ago, various researches have been conducted to study and explore the active components which are responsible for these claimed benefits. These components are the quassinoids for example eurycomanone [3, 4, 7], canthine-6-one alkaloids [4, 8–12], squalene derivatives [13], and byphenylneolignans [14]. Considering the fact that this herbal extract increased the level of testosterone in sexually insufficient individuals, there was an effort to explore the role of *Eurycoma longifolia *in treatment of androgen-deficiency-related diseases. An earlier study observed the potential of *Eurycoma longifolia* for the treatment of androgen-deficient osteoporosis [15].

Osteoporosis is a systemic skeletal disease characterized by low bone mass and microarchitectural deterioration of bone tissue with a consequent increase in bone fragility and increased fracture risk [16]. Male osteoporosis which was not recognized earlier has now become an important public health issue. Although hip fractures involve less than one-third of males, the mortality rate has been reported to be 31% and 17% in males and females, respectively [17]. Osteoporotic fracture in males is associated with significant morbidity and mortality, which have huge emotional and financial impacts on their families and the society [18].

The treatment of choice for men with androgen-deficient osteoporosis is testosterone-replacement therapy. This therapy is recommended based on the increasing understanding on the role of androgens in bone physiology and remodeling, which is made evident by numerous animal and human studies [19]. However, testosterone replacement therapy is contraindicated in patients with prostate or breast cancer [20]. Some physicians even perceived that testosterone replacement therapy is associated with increased risk of prostate cancer. A study reported that almost 35% of hypogonadal men did not receive treatment due to fear of developing prostate cancer [21]. Another contraindication which is worth mentioning is sleep apnoea as hypogonadal patients may have worsening of sleep apnoea with testosterone replacement [20]. Some may also relate testosterone replacement therapy with cardiovascular disease, liver damage, and erythrocytosis [22]. Thus, it is necessary and essential to find an alternative treatment which not only gives protection to the bone but at the same time possesses fewer side effects compared to the testosterone therapy.

Over the past few decades, *Eurycoma longifolia* has been recognized internationally especially as an aphrodisiac and is being used widely to enhance virility and sexual performance in male [23, 24]. Many studies reported that this ability of *Eurycoma longifolia* is associated with the increased level of testosterone in subjects supplemented with *Eurycoma longifolia*. Recent human trials showed that *Eurycoma longifolia* supplementation increased the level of testosterone, together with the increase in the superoxide dismutase (SOD), an antioxidant which plays an important role in slowing down the process of aging [25–28]. Despite the documented history of *Eurycoma longifolia* as a testosterone-raising herb, the underlying mechanisms for its androgenic effects remain unclear. Shawn and Kraemer (2007) in their study suggested that *Eurycoma longifolia* increased testosterone levels by way of promoting the dissociation of testosterone from sex-hormone-binding globulin (SHBG) [29]. This SHBG is known to increase in aging men and causes a decrease in the bioavailability of the active fractions of testosterone. One of the bioactive components of *Eurycoma longifolia*, eurypeptide may enhance the biosynthesis of different androgens, by activating the CYP17 (17 *α*-hydroxylase) enzyme, thereby boosting further the metabolism of pregninolone and 17-OH-progesterone to dehydroepiandrosterone (DHEA) [30]. It also enhances the metabolism of progesterone and 17-OH-progesterone to 4-androstenedione and testosterone [30].

The World Health Organization (WHO) defines osteoporosis as bone mineral density (BMD) value of 2.5 standard deviations or more below the young adult women mean value [31]. BMD measurement by dual energy X-ray absorptiometry (DEXA) is currently the most common technique for assessing the risk of osteoporosis. However, this BMD measurement may not reflect the actual bone strength and the risk of fragility fracture. In a prospective study involving 699 subjects, it was observed that there was an overlapping of BMD measurements between subjects with and without fractures [32]. In the same study, they found that the relationship between bone mass and fracture risk was not linear. The trabecular bone, which is predominantly affected in osteoporotic changes, is a continuous three-dimensional network of bars and plate, with varying densities and orientations. Several studies have suggested that connectivity density of the trabecular bone, rather than bone density, may be the parameter mostly affected by osteoporotic changes [33–35]. Connectivity density, with regard to bones microarchitecture, is defined as the maximal number of branches that can be broken before the microarchitecture is separated into parts [36]. Apart from trabecular bone density, histomorphometry analysis of the bone microarchitecture also contributes to the prediction of fracture risk [33, 37]. However, this conventional and static two-dimensional histomorphometry gives only limited information on bone microarchitecture [38]. A three-dimensional, nondestructive method of bone microarchitecture measurement pioneered by Feldkamp et al. in 1989, has become feasible only in recent years [39]. This microcomputed tomography (micro-CT) imaging system gives a detailed picture of three-dimensional bone architecture together with bone connectivity density. This high-resolution imaging technique has become more popular and largely applied in the field of bone research, both in basic as well as preclinical. Thus in this study, we aimed to observe the effects of *Eurycoma longifolia* supplementation on the trabecular bone microarchitecture in orchidectomised rats using micro-CT.

## 2. Materials and Methods

### 2.1. Experimental Animals and Treatment

Forty-eight *Sprague Dawley* rats aged 10–12 months old (300–400 mg) were divided into six groups of sham-operated (SHAM), orchidectomised control (ORX), orchidectomised + testosterone enanthate 7 mg/rat (TEN), orchidectomised + *Eurycoma longifolia* 30 mg/kg (EL30), orchidectomised + *Eurycoma longifolia* 60 mg/kg (EL60) and orchidectomised + *Eurycoma longifolia *90 mg/kg (EL90). *Eurycoma longifolia* standardized aqueous extract (EL) was given 6 days a week via oral gavages, while testosterone was injected intramuscularly weekly throughout the six weeks duration of the study. The rats were housed singly in plastic cages at room temperature with a 12-hour light-dark cycle. They were fed with commercial rat chow (Gold Coin, Selangor, Malaysia) and tap water *ad libitum.* Ethical approval was obtained from the Universiti Kebangsaan Malaysia Animal Ethics Committee (UKMAEC) (PP/FAR/2011/NAZRUN/22-MARCH/362-JUNE-2011-MAY-2012).

### 2.2. Materials and Bone Preparation

*Eurycoma longifolia* standardized aqueous extract (EL) was obtained from Phytes Biotek Sdn, Bhd. (Shah Alam, Malaysia). The extract was in brownish powder form and the bioactive components were eurypeptide (22.0%), glycosaponin (41.1%) and eurycomanone (1.6%). EL aqueous powder was dissolved in deionized water and given via oral gavages at doses of 30, 60, and 90 mg/kg rat weight at 9 am six days a week for six weeks [40]. Testosterone enanthate (Jesalis Pharma, Germany) was diluted in peanut oil and 7 mg/rat was administered via intramuscular injection once a week throughout the study period [41]. At the end of the treatment, the rats were euthanized by overdose of diethyl ether. Femora were dissected and cleaned from all soft tissues. They were then stored in 10% formalin solution until analyzed.

### 2.3. Micro-CT Analysis

The effect of EL supplementation on trabecular bone was assessed using micro-CT (*μ*CT80 scanner, Scanco Medical, Switzerland). Before scanning, measurement protocols to define parameters such as source energy and image resolution were created. The left femur was placed in a sample holder in a vertical direction with the epiphyseal head facing downward. The source energy selected for this study were 70 KVp and 114 *μ*A with image resolution set as “high” to obtain the best contrast between bone and soft tissues. The trabecular bone parameters were obtained from the distal end of the left femur. Scanning was done at the metaphyseal area located 1.5 mm below the lowest point of the epiphyseal growth plate and extending 2.0 mm in the proximal direction. This is the secondary spongiosa area, which is rich in high-turnover trabecular bone. Trabecular bone was chosen because its remodelling process is more dynamic than the cortical bone [42].

### 2.4. Statistical Analysis

The results were expressed as mean ± standard error of the mean (SEM). The data analysis was performed using the Statistical Package for Social Sciences software (SPSS 19.0; SPSS, Chicago, IL, USA). The data were tested for normality using the Kolmogorov-Smirnov test. For normally distributed data, the statistical tests used were the analysis of variance (ANOVA), followed by Tukey's Honestly Significant Difference (HSD) test. For data that were not normally distributed, Kruskal-Wallis and Mann-Whitney tests were used.

## 3. Results

**Figure 1:**
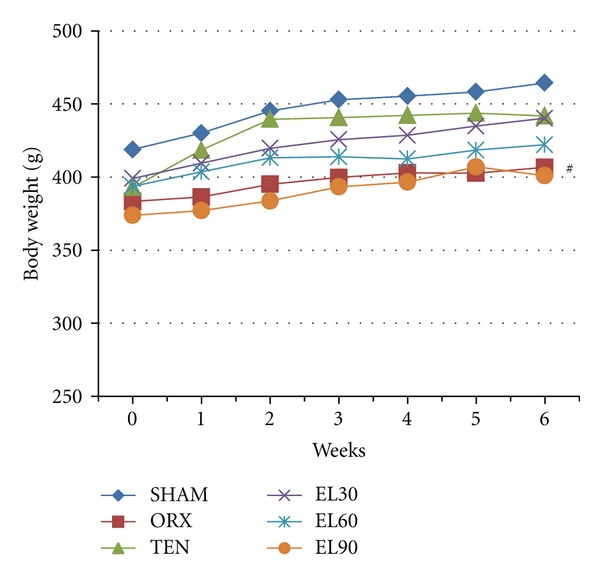
Mean body weight throughout the study. SHAM, sham-operated; ORX, orchidectomised-control; TEN, orchidectomised + 7 mg/rat testosterone enanthate; EL30, orchidectomised + *Eurycoma longifolia* 30 mg/kg; EL60, orchidectomised + *Eurycoma longifolia* 60 mg/kg; EL90, orchidectomised + *Eurycoma longifolia* 90 mg/kg.

There were no significant differences in body weight between the different groups at the beginning of the study. The body weight increased steadily throughout the period of the study. At the end of the study, there was a significant decrease in the body weight of the ORX group compared to the SHAM group (*P* < 0.01). The body weights of all the other groups showed no significant difference compared to each other (Figure 1).

**Figure 2:**
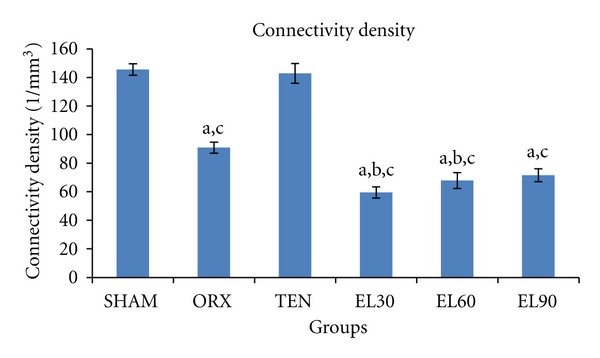
Trabecular bone connectivity density for all the groups after 6 weeks of treatment. SHAM, sham-operated; ORX, orchidectomised-control; TEN, orchidectomised + 7 mg/rat testosterone enanthate; EL30, orchidectomised + *Eurycoma longifolia* 30 mg/kg; EL60, orchidectomised + *Eurycoma longifolia* 60 mg/kg; EL90, orchidectomised + *Eurycoma longifolia* 90 mg/kg. ^a^ *P* < 0.05 versus SHAM, ^b^ *P* < 0.05 versus ORX, ^c^ *P* < 0.05 versus TEN.

In the present study, orchidectomy significantly decreased (−37.56%, *P* < 0.05) the trabecular bone connectivity density compared to the SHAM group (Figure 2). In contrast, the group supplemented with testosterone enanthate (TEN) showed an increase (+57.30%, *P* < 0.05) in trabecular bone connectivity density compared to the ORX group. Generally, the groups supplemented with EL showed a dose-dependent increase in the trabecular bone connectivity density. However, the bone connectivity density of these groups were significantly lower (*P* < 0.05) than the SHAM, ORX, and TEN groups. Only the group with the highest dose of EL (EL90) showed similar trabecular bone connectivity density compared to that of the ORX group.

**Figure 3:**
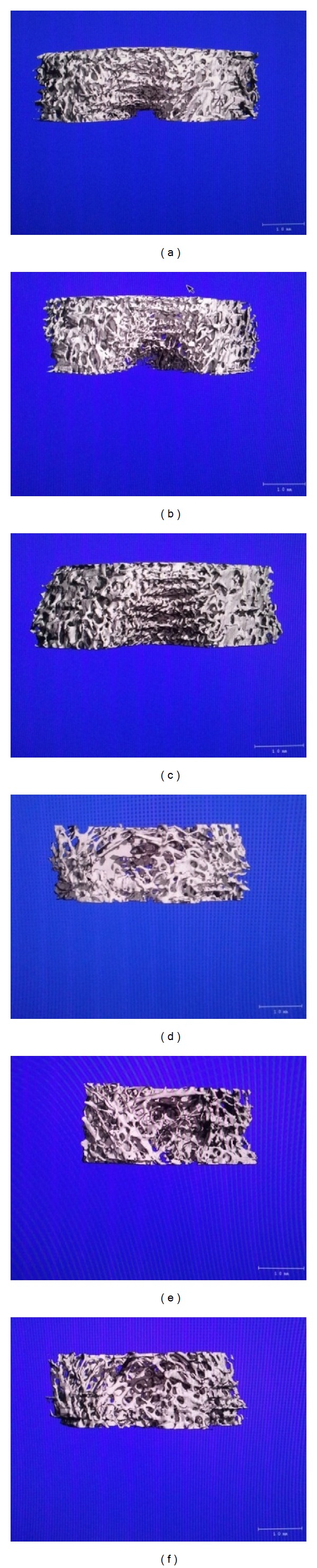
Three dimensional micro-CT images of the trabecular microstructure of distal femur metaphysis. (a) sham-operated; (b) orchidectomised-control; (c) orchidectomised + 7 mg/rat testosterone enanthate; (d) orchidectomised + *Eurycoma longifolia* 30 mg/kg; (e) orchidectomised + *Eurycoma longifolia* 60 mg/kg; (f) orchidectomised + *Eurycoma longifolia* 90 mg/kg.

Figure 3 depicts the three-dimensional image of the distal femur metaphysis. The bone tissue chosen as the region of interest (ROI) shown in white has been reconstructed in three dimension to see the structural context of trabecular bone. The loss of trabecular bone connectivity density is apparent in the groups treated with EL (EL30, EL60 and EL90) compared to the SHAM and TEN groups, with associated changes in other indices of trabecular bone microarchitecture.

**Figure 4:**
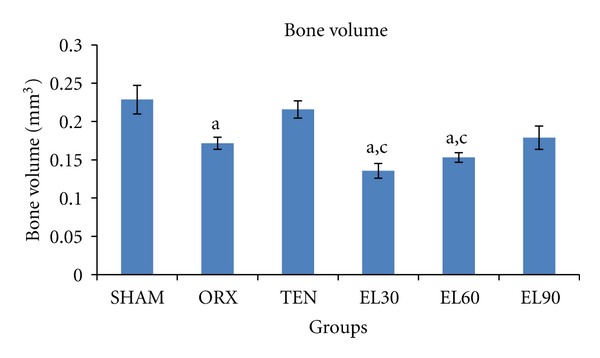
Bone volume for all the groups after 6 weeks of treatment. SHAM, sham-operated; ORX, orchidectomised control; TEN, orchidectomised + 7 mg/rat testosterone enanthate; EL30, orchidectomised + *Eurycoma longifolia* 30 mg/kg; EL60, orchidectomised + *Eurycoma longifolia* 60 mg/kg; EL90, orchidectomised + *Eurycoma longifolia* 90 mg/kg. ^a^ *P* < 0.05 versus SHAM, ^b^ *P* < 0.05 versus ORX, and ^c^ *P* < 0.05 versus TEN.

Bone volume was significantly decreased in the ORX, EL30 and EL60 groups (−24.97, −40.72, and −33.11%, resp., *P* < 0.05 for all) compared to the SHAM group (Figure 4). EL supplementation at 90 mg/kg was effective in preserving bone volume as it showed no significant difference compared to the SHAM group.

**Figure 5:**
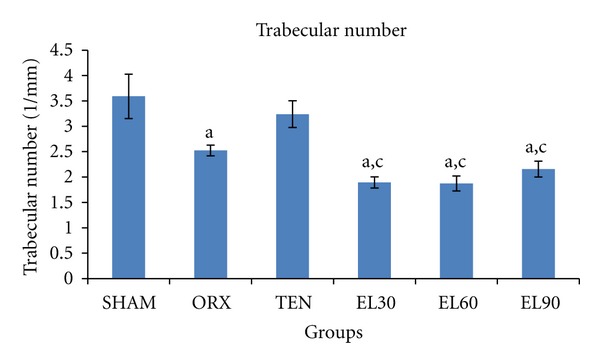
Trabecular number for all the groups after 6 weeks of treatment. SHAM, sham-operated; ORX, orchidectomisedcontrol; TEN, orchidectomised + 7 mg/rat testosterone enanthate; EL30, orchidectomised + *Eurycoma longifolia* 30 mg/kg; EL60, orchidectomised + *Eurycoma longifolia* 60 mg/kg; EL90, orchidectomised + *Eurycoma longifolia* 90 mg/kg. ^a^ *P* < 0.05 versus SHAM, ^b^ *P* < 0.05 versus ORX, and ^c^ *P* < 0.05 versus TEN.

**Figure 6:**
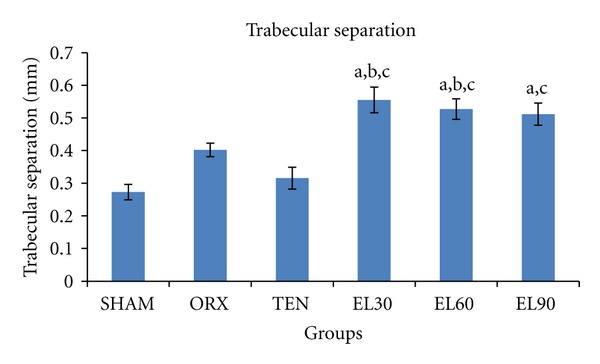
Trabecular separation for all the groups after 6 weeks of treatment. SHAM, sham-operated; ORX, orchidectomised control; TEN, orchidectomised + 7 mg/rat testosterone enanthate; EL30, orchidectomised + *Eurycoma longifolia* 30 mg/kg; EL60, orchidectomised + *Eurycoma longifolia* 60 mg/kg; EL90, orchidectomised + *Eurycoma longifolia* 90 mg/kg. ^a^ *P* < 0.05 versus SHAM, ^b^ *P* < 0.05 versus ORX, ^c^ *P* < 0.05 versus TEN.

There was a significant decrease in the trabecular number in the ORX and all EL treated groups (−29.77, −47.23, −47.79, and −39.91%, resp., *P* < 0.05 for all) compared to the SHAM group (Figure 5). Similarly, all EL treated groups showed significantly higher trabecular separation (+103.36, +92.96 and +87.36%, resp., *P* < 0.05 for all) compared to the SHAM group (Figure 6).

**Figure 7:**
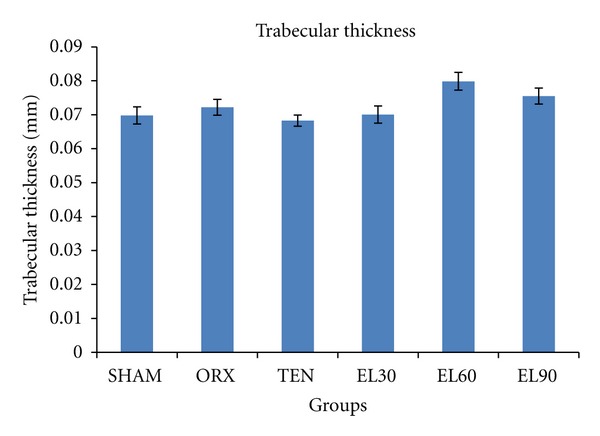
Trabecular thickness for all the groups after 6 weeks of treatment. SHAM, sham-operated; ORX, orchidectomised control; TEN, orchidectomised + 7 mg/rat testosterone enanthate; EL30, orchidectomised + *Eurycoma longifolia* 30 mg/kg; EL60, orchidectomised + *Eurycoma longifolia* 60 mg/kg; EL90, orchidectomised + *Eurycoma longifolia* 90 mg/kg. ^a^ *P* < 0.05 versus SHAM, ^b^ *P* < 0.05 versus ORX, and ^c^ *P* < 0.05 versus TEN.

As for the trabecular thickness parameter, there were no significant differences between the groups (Figure 7).

## 4. Discussion

In males, androgen deficiency caused severe loss of bone [43], muscle, and fat mass [44, 45]. These may have contributed to the low body weight of rats in the orchidectomised control group. The body weights of rats supplemented with EL or receiving testosterone were preserved from the weight loss effects of orchidectomy. This may be due to the anabolic effects of testosterone and EL on the bone and body compositions. Androgen replacement therapy on both orchidectomised rats and hypogonadal men were found to reverse the effects of androgen deficiency on muscle mass [45–47]. The androgenic effect of EL used in this study was supported by a five-week human study, which showed that supplementation of water soluble extract of *Eurycoma longifolia* increased muscle mass and strength [48].

In the present study, the trabecular bone microarchitecture was assessed using the state of the art micro-CT. It is capable of three-dimensional bone analysis which produces high quality images, thus well accepted by the scientific communities. A comparison study revealed that all radiographic parameters (bone thickness and length) were significantly correlated to the corresponding micro-CT measurements [49]. Another study comparing three-dimensional micro-CT and two-dimensional histomorphometry, found good correlations between these two methods [50]. The same study also showed an excellent correlation in the connectivity density estimation using ConnEuler principle and micro-CT.

As expected, the present study showed that androgen withdrawal induced by orchidectomy had caused significant deteriorations of the bone microarchitecture of the rats at six weeks post-orchidectomy. These findings were supported by Yao et al. (2005) who reported that orchidectomy caused reduction in trabecular bone connectivity density but caused no change in trabecular thickness [51]. Meanwhile, studies by Yarrow et al. (2008) and Libouban et al. (2008) showed that orchidectomy caused deterioration in all trabecular bone microarchitectures which include trabecular bone number, thickness and separation [41, 52]. Testosterone replacement was able to reverse all the orchidectomy-induced changes. This was in agreement with Yarrow et al. (2008) which showed that testosterone administration to orchidectomised rats significantly improved the trabecular number, width and separation [41].

In a previous study, EL supplementation at 15 mg/kg was able to prevent bone calcium loss in orchidectomised rats, although not as effective as testosterone treatment [15]. In the present study, higher EL doses of 30, 60, and 90 mg/kg were used to determine whether EL supplementations would affect bone microarchitecture in a dose-dependent manner.

Surprisingly, data from this study showed that EL at doses of 30 and 60 mg/kg failed to protect bone from orchidectomy-induced changes. Supplementation of 90 mg/kg EL showed some protection on the bone volume. Meanwhile, EL doses of 30 and 60 mg/kg worsened all the bone microarchitecture indices such as the bone connectivity density, volume, number, and separation. There is paucity of literature on the effects of *Eurycoma longifolia* on osteoporosis. However, it can be postulated that EL may directly damage bone cells or they may be involved in the regulation of osteoblasts and osteoclasts activities, resulting in increased bone resorption. The standardized water extract of EL used in this study contains eurycomanone, which was found to be toxic to human cells [10]. However, *in vitro *toxicity studies often used methanolic root extracts of *Eurycoma longifolia* [10, 53], which is more potent, and thus may explain its toxic effects on human cells. Apart from eurycomanone, there are other *Eurycoma longifolia* constituents, which were shown to possess cytotoxic activities such as *β* carboline alkaloid, which was active against human lung and breast-cancer cells and canthine-6-1 alkaloid, which was active against multiple human cancerous cells [1, 10, 54]. The toxic effects of these EL constituents may have caused the deterioration of the bone microarchitecture found in this study.

Bone is composed of supporting cells (osteoblast and osteocytes), remodeling cells (osteoclasts) osteoid and inorganic mineral salts (hydroxyapatite). Osteoblasts synthesize new collagenous matrix and regulate its mineralization by concentrating calcium and phosphate, and destroying mineralization inhibitors such as pyrophosphate and proteoglycan [55, 56]. Approximately 30 to 50% of osteoblasts become osteocytes or bone-lining cells, while the majority of them undergo apoptosis [57]. The bone-lining cells differentiate into osteoblasts upon exposure to parathyroid hormone or mechanical forces [58]. The formation, activation, and resorption of osteoclasts, the bone-resorbing cells, are regulated by the ratio of receptor activator of NF-*κβ* ligand (RANKL) to osteoprotegerin (OPG), Interleukin-1 (IL-1) and Interleukin-6 (IL-6), macrophage colony stimulating factor (M-CSF), parathyroid hormone, 1,25-dihydroxyvitamin D, and calcitonin [59, 60]. Both RANKL and M-SCF are produced by osteoblasts and marrow stromal cells, and the presence of these two cells is very important in osteoclastogenesis [61, 62]. Bone remodeling is the combination of bone resorption and formation by osteoclasts and osteoblasts, respectively, to maintain bone mass and bone strength, which can be assessed by measuring the bone microarchitecture indices. Thus, any interruption to these cytokines or bone cells regulating the activities of bone resorption and bone formation, will affect these measurements. The present study showed that EL30 and EL60 groups have suffered deterioration in the bone microarchitectures. Studies have shown that estrogen withdrawal following ovariectomy caused deterioration of trabecular bone microarchitecture which was related to perforation of trabecular plates and loss of connectivity density [63–65]. In the present study, this loss of connectivity density was reversible only with testosterone replacement but not with EL30 and EL60 supplementations. A study by Yao et al. (2005) on the long bone (tibia) reported that ovariectomy caused reduction in total trabecular bone volume, connectivity, and trabecular number while trabecular thickness did not change significantly [51]. In the current study, testosterone depletion following orchidectomy showed similar results. Yao et al. (2005) also reported that connectivity density of the tibia showed contradictory findings compared to the vertebra, partly due to the slower bone loss rate in the vertebra than in the tibia: there was only 20% loss of trabecular bone in the vertebra four months postovariectomy versus more than 80% in the tibia [51]. In the vertebra, estrogen depletion-induced fenestration of plates, which increased the connectivity density, and treatments with anabolic or antiresorptive agents filled in the fenestration, resulting in a lower connectivity density of the bone [51].

There were no significant changes in the trabecular thickness for all the groups. Physiologically, with the loss of bone connectivity density, reduction in trabecular number and widening of the trabecular separation, there would be a compensatory trabecular thickening. This finding is in agreement with Gasser et al. (2005) who found that zoledronic acid (antiresorptive agent) increased trabecular thickness without improving the bone connectivity density [66]. Other studies have also reported compensatory increase in the remaining trabecular following the loss of bone mass during adulthood [67–70]. This compensatory mechanism may account for the nonsignificant changes in the trabecular thickness for the orchidectomised rats seen in the present study.

 Supplementation of EL at 90 mg/kg showed preservation in the trabecular volume although it was less effective than testosterone. As mentioned earlier, EL may have indirectly improved bone volume by increasing the testosterone level. Although there is paucity in literature on the effects of EL on bone microarchitecture, there are many studies on the relationship between testosterone and bone metabolism and structure. A study on hypogonadal men given testosterone treatment showed significant increases in trabecular bone volume fraction and thickness after 24 months of treatment [71]. Meanwhile, another study on supraphysiological administration of testosterone enanthate also showed improvements on the trabecular number and width, and reduction in trabecular separation [41].

Although the exact mechanisms are still unclear, it was suggested that *Eurycoma longifolia* increased free testosterone level by enhancing the dissociation of testosterone from SHBG [29]. *Eurycoma longifolia* has also been shown to enhance the metabolism of different types of androgens, thus increasing the levels of more biologically active androgens, testosterone, and DHEA [30]. The latter mechanism may be supported by recent human trials on *Eurycoma longifolia* supplementation, which showed that water extract of *Eurycoma longifolia* increased the total testosterone and DHEA levels [25–28, 72]. In animal studies involving rats and mice, both the water and ethanolic extracts of *Eurycoma longifolia* were found to increase the sexual motivation, performance, and frequency of sexual activity [2, 23]. In one of the studies, it was shown that at 800 mg/kg, *Eurycoma longifolia* promoted the growth of the reproductive organs (seminal vesicles and ventral prostate) in male rats [23]. These effects resembled those induced by testosterone administration [73]. Testosterone may exert its effects directly via activation of the androgen receptor (AR) found on bone skeletal surfaces or indirectly via aromatization of androgens (testosterone) to estrogen [19]. Thus, the increase in androgens may play an important role in maintaining the bone volume of the rats supplemented with 90 mg/kg dose of EL.

## 5. Conclusion

In conclusion, EL supplementations at the dose of 30 and 60 mg/kg deteriorated the bone microarchitecture of orchidectomised rats. At higher dose of 90 mg/kg, EL supplementation was only able to preserve bone volume. Further studies are required to determine if low dose of EL is toxic to bone cells, and if higher dose than 90 mg/kg would provide better protection to bone microarchitecture.
